# Supplementary material for: Oxygen as a Possible Technological Adjuvant during the Crushing or the Malaxation Steps, or Both, for the Modulation of the Characteristics of Extra Virgin Olive Oil
Source: Foods. 2023 May 27;12(11):2170. doi: 10.3390/foods12112170 (PMC10252381; doi:10.3390/foods12112170)
Supplement: Supplementary file 1 [file foods-12-02170-s001.zip › foods-2391972-supplementary.pdf]

Tabla S1. Metabolites identified at different level according to their chemical group.

Metabolite	Group label	Level
**3-Methyl-furan**	Furan	2
**3-Methyl-butanal**	Adehyde no LOX	1
**2-Methyl-butanal**	Adehyde no LOX	1
**Octanal**	Adehyde no LOX	1
**Nonanal**	Aldehyde no LOX	1
**(E,E)-2,4-Hexadienal**	Adehyde no LOX	1
**Benzaldehyde**	Adehyde no LOX	1
**(Z)-2-Heptenal**	Adehyde no LOX	1
**Methanol**	Alcohol no LOX	1
**Ethanol**	Alcohol no LOX	1
**(Z)-2-Hepten-1-ol**	Alcohol no LOX	2
**Benzyl alcohol**	Alcohol no LOX	1
**4-Methyl-2-pentanone**	Ketone no LOX	2
**6-Methyl-5-hepten-2-one**	Ketone no LOX	1
**2-Octanone**	Ketone no LOX	2
**Ethyl butyrate**	Ester no LOX	2
**Butyl acetate**	Ester no LOX	2
**Ethyl isovalerate**	Ester no LOX	2
**Toluene**	HC aromatic	2
**Ethylbenzene**	HC aromatic	1
**α-Ocimene**	HC-monoterpene	1
**(E)-4,8-Dimethylnona-1,3,7-triene**	HC-terpene	2
**UK-1**	UK-1	4
**UK-2**	UK-2	4
**Hexanal**	Aldehyde LOX	1
**(Z)-2-Pentenal**	Aldehyde LOX	1
**(E)-2-Pentenal**	Aldehyde LOX	1
**(Z)-3-Hexenal**	Aldehyde LOX	1
**(E)-3-Hexenal**	Aldehyde LOX	1
**(Z)-2-Hexenal**	Aldehyde LOX	1
**(E)-2-Hexenal**	Aldehyde LOX	1
**1-Penten-3-ol**	Alcohol LOX	1
**1-Hexanol**	Alcohol LOX	1
**(Z)-3-Hexen-1-ol**	Alcohol LOX	1
**Hexyl acetate**	Ester LOX	1

**(Z)-3-Hexenyl acetate**	Ester LOX	1
**Pentene dimers**	LOX hydrocarbon	2
**2-Pentanone**	Ketone LOX	1
**3-Pentanone**	Ketone LOX	1
**1-Penten-3-one**	Ketone LOX	1
**4-Methyl-2-pentanol**	Internal Standard	1
